# Serum Galactose-Deficient IgA1 Level Is Not Associated with Proteinuria in Children with IgA Nephropathy

**DOI:** 10.1155/2012/315467

**Published:** 2012-06-17

**Authors:** M. Colleen Hastings, Sabahat Afshan, John T. Sanders, Oulimata Kane, T. Matthew Eison, Keith K. Lau, Zina Moldoveanu, Bruce A. Julian, Jan Novak, Robert J. Wyatt

**Affiliations:** ^1^Children's Foundation Research Institute, Le Bonheur Children's Hospital, 50 North Dunlap, Memphis, TN 38103-2893, USA; ^2^Department of Pediatrics, University of Tennessee Health Science Center, 50 North Dunlap, Memphis, TN 38103, USA; ^3^Department of Medicine, University of Tennessee Health Science Center, 956 Court Avenue, Memphis, TN 38163, USA; ^4^Sanford Children's Hospital Sioux Falls, 1305 W 18th Street, Sioux Falls, SD 57117-5039, USA; ^5^Department of Pediatrics, McMaster University, 1280 Main Street, West Hamilton, ON, Canada L8S 4K1; ^6^Department of Microbiology, University of Alabama at Birmingham, 845 19th Street South, Birmingham, AL 35294, USA; ^7^Division of Nephrology, Department of Medicine, University of Alabama at Birmingham, 1530 Third Avenue South, Birmingham, AL 35294, USA

## Abstract

*Introduction*. Percentage of galactose-deficient IgA1 (Gd-IgA1) relative to total IgA in serum was recently reported to correlate with proteinuria at time of sampling and during follow-up for pediatric and adult patients with IgA nephropathy. We sought to determine whether this association exists in another cohort of pediatric patients with IgA nephropathy. *Methods*. Subjects were younger than 18 years at entry. Blood samples were collected on one or more occasions for determination of serum total IgA and Gd-IgA1. Gd-IgA1 was expressed as serum level and percent of total IgA. Urinary protein/creatinine ratio was calculated for random specimens. Spearman's correlation coefficients assessed the relationship between study variables. *Results*. The cohort had 29 Caucasians and 11 African-Americans with a male : female ratio of 1.9 : 1. Mean age at diagnosis was 11.7 ± 3.7 years. No statistically significant correlation was identified between serum total IgA, Gd-IgA1, or percent Gd-IgA1 versus urinary protein/creatinine ratio determined contemporaneously with biopsy or between average serum Gd-IgA1 or average percent Gd-IgA1 and time-average urinary protein/creatinine ratio. *Conclusion*. The magnitude of proteinuria in this cohort of pediatric patients with IgA nephropathy was influenced by factors other than Gd-IgA1 level, consistent with the proposed multi-hit pathogenetic pathways for this renal disease.

## 1. Introduction

IgA nephropathy (IgAN) is the most common form of chronic glomerulonephritis for individuals of European and Asian descent [1, 2]. The level of proteinuria at diagnosis of IgAN has been associated with the primary endpoint of outcome (i.e., progression to chronic dialysis or transplantation) in adults [3–7] and children [8–11].

Data from clinical and basic research in IgAN has led to the hypothesis that four hits are responsible for clinical expression of IgAN [12]. The first hit is the presence of aberrantly glycosylated *O*-linked glycans on the heavy-chain hinge region of circulatory IgA1 that terminate in *N*-acetylgalactosamine (GalNAc) rather than galactose [13]. Elevated serum levels of this galactose-deficient IgA1 (Gd-IgA1) were found in 76% of 153 Caucasian adults with IgAN in the United States [14]. IgAN patients in Japan and China also had elevated serum Gd-IgA1 levels [15, 16], as did African-American patients in the southeastern United States [17]. In addition, elevated serum Gd-IgA1 levels were found in 77% of 22 African-American and Caucasian children with IgAN [18].

The second hit is the induction of circulating IgG or IgA antibodies specific for Gd-IgA1 [19], and the third hit is the resultant formation of nephritogenic immune complexes and their deposition in the glomerular mesangium. The final hit is the induction by these immune complexes of a local proliferative and inflammatory response of the mesangial cells [20–22]. Logically, the events related to the fourth hit would be directly or indirectly responsible for the induction of proteinuria in IgAN. In a recent study, the percentage of serum Gd-IgA1 relative to serum total IgA1 was found to correlate with proteinuria at time of sampling and over the follow-up interval in 62 pediatric and adult patients [23]. Those data differed from the findings at the time of sampling in our initial report with adult patients [14]. The aim of the present study was to determine whether the serum level of Gd-IgA1 associates with proteinuria in a well-characterized cohort of pediatric patients with IgAN.

## 2. Patients and Methods

### 2.1. Study Population

The subjects had been entered into earlier studies approved by the Institutional Review Boards of the University of Tennessee Health Science Center and the University of Alabama at Birmingham. The diagnosis of IgAN was established by renal biopsy showing IgA as the dominant or codominant immunoglobulin in a typical mesangial distribution, in the absence of clinical and laboratory evidence for systemic disease [24]. All subjects were younger than 18 years of age at the time of diagnostic biopsy. This study did not enroll children who had received a kidney transplant or who were on dialysis. Data for initial serum Gd-IgA1 level were previously reported for 22 of the patients included in the present report [17]. Of the 40 patients in this study, 31 were diagnosed and followed up by the pediatric nephrology group at the Le Bonheur Children's Hospital (LBCH) in Memphis, TN (LBCH cohort), five were diagnosed at other centers and seen in consultation at LBCH, two were diagnosed and followed in Lexington, KY, and two in Birmingham, AL.

Serum samples were available from 97 healthy controls younger than 18 years. The control group included 29 African-American males, 21 African-American females, 28 Caucasian males, and 19 Caucasian females.

### 2.2. Laboratory Measures and Data Collection

Blood samples were collected from patients on one or more occasions for determination of serum total IgA, Gd-IgA1, and creatinine concentration. Urinary protein and creatinine concentrations were measured in the clinical laboratory from a random spot urine sample, and a urinary protein/creatinine ratio (UPCR) (g/g) was calculated. Estimated GFR was calculated with the new Schwartz formula [25]. Systolic and diastolic blood pressure percentiles based upon age, gender, and height percentile were determined by the tables from the Fourth Report on the Diagnosis, Evaluation, and Treatment of High Blood Pressure in Children and Adolescents [26]. The blood pressure used for this calculation was the average of available measurements within two months of biopsy, if more than one was recorded. BMI percentile was determined using the QuesGen Systems, Inc. web-based calculator that used National Health and Nutrition Examination Survey data as the source for calculations (http://www.quesgen.com/BMIPedsCalc.php).

Time-average (TA) proteinuria was determined according to the description of Reich et al. [7], except that UPCR was used instead of results of timed urine collections. The UPCR was determined for each six-month interval after biopsy; if there were two or more values for an interval, the mean of the values was used. The TA-UPCR was derived by averaging these UPCRs from each six-month interval of follow-up time.

Serum total IgA and Gd-IgA1 levels were determined by ELISA, as described previously [14]. The Gd-IgA1 ELISA used biotinylated lectin from *Helix aspersa* (Sigma-Aldrich, St. Louis, MO, USA) that binds specifically to terminal GalNAc. Two galactose-deficient IgA1 myeloma proteins, McE and Ale, were used as standards in the Gd-IgA1 assays. Results for levels of Gd-IgA1 were expressed as U/mL serum, with 1 U (unit) corresponding to 1 *μ*g of Gd-IgA1 myeloma standard protein. During the course of the study, the standard in the assay was changed from McE to Ale; the latter IgA1 myeloma protein has a slightly higher content of terminal GalNAc. The McE standard had been used exclusively for our initial reports [14, 17]. Subsequent levels determined using the Ale standard were multiplied by a factor of 2.5 to be compared to those determined in assays using the McE protein as standard.

### 2.3. Statistical Analyses

 The Mann-Whitney *U* test was used to determine differences between patient and control groups for serum levels of Gd-IgA1 and percent Gd-IgA1/IgA. Spearman's correlation coefficients were used to assess the relationship between study variables. SAS 9.1 (SAS Institute, Cary, NC, USA) was used for descriptive statistics and calculation of correlation coefficients.

## 3. Results

Incident cases are defined as those having a serum Gd-IgA1 level first measured within 3 months after the diagnostic biopsy. Prevalent cases had their first Gd-IgA1 measurement after longer intervals. Clinical and demographic data are shown in Table 1 for the incident cases and in Table 2 for the prevalent cases.

The LBCH cohort differed from the other cases because of higher percentages of African-Americans and subjects with TA-UPCR determinations Table 3. The 40 patients included 29 Caucasians and 11 African Americans; 26 were male. For the IgAN group, the mean age ± SD at diagnosis was 11.7 ± 3.7 years and 13.2 ± 3.7 years at the time the first serum for Gd-IgA1 level was obtained. The mean ± SD age at time of study was 12.6 ± 2.9 years for the healthy control group.

Measurements of serum Gd-IgA1 and UPCR on the same date were available on at least one occasion for 40 patients; 13 patients had from two to four paired samples over the course of observation. A total of 62 paired samples for serum Gd-IgA1 and UPCR were available for analysis.

**Figure 1:**
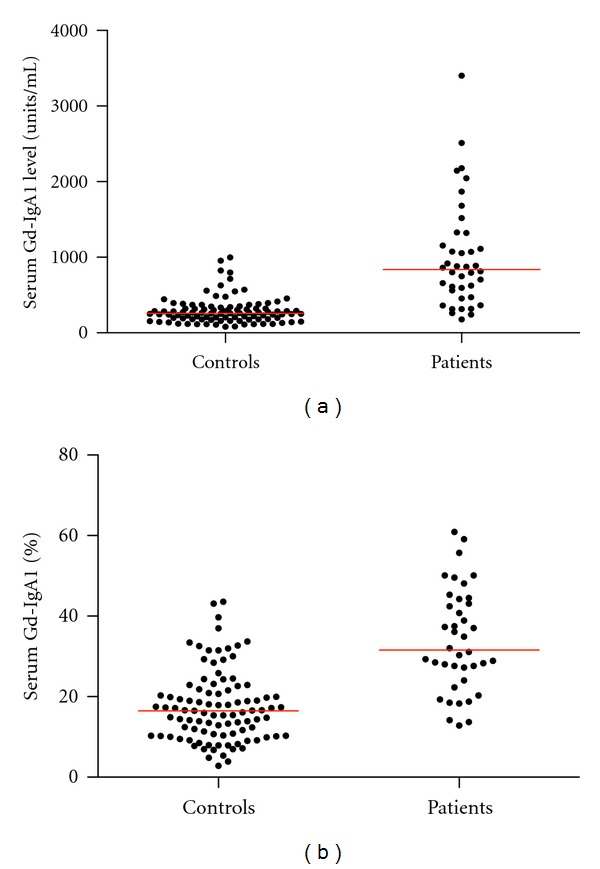
Serum levels of Gd-IgA1 plotted for the initial sample for 40 pediatric patients and 97 healthy pediatric controls. (a) Represented as units/mL serum. Median is represented for each group. The serum level was significantly higher for the patient group (*P* < 0.0001). (b) Represented as percentage of total IgA. Median is represented for each group by the red bar. The serum level was significantly higher for the patient group (*P* < 0.0001).

The initial serum Gd-IgA1 levels for the patient and control groups are shown in Figure 1(a). The patient group levels were significantly higher than those of the control group (*P* < 0.0001). The median serum Gd-IgA1 level for 97 healthy controls under age 18 years was 260 U/mL (interquartile range (IQR) 183–334 U/mL). Based upon these controls, the 90th and 95th percentiles were 482 U/mL and 645 U/mL, respectively. For patients, median serum Gd-IgA1 level was 688 U/mL (IQR 517–1238 U/mL) and the median total serum IgA level was 2499 U/mL (IQR 1930–4072 U/mL).

The initial medians for percent Gd-IgA1/total serum IgA for the patient and control groups are shown in Figure 1(b). The patient group levels were significantly higher than those of the control group (*P* < 0.0001). The median percent Gd-IgA1/total serum IgA level for 97 healthy controls under age 18 years was 17%, with an IQR of 10% to 22%. Based upon these controls, the 90th and 95th percentiles were 32% and 34%, respectively. For patients, initial median percent Gd-IgA1/total serum IgA was 32% (IQR 26%–48%). The median UPCR was 0.74 g/g (IQR 0.23–1.68 g/g).

**Figure 2:**
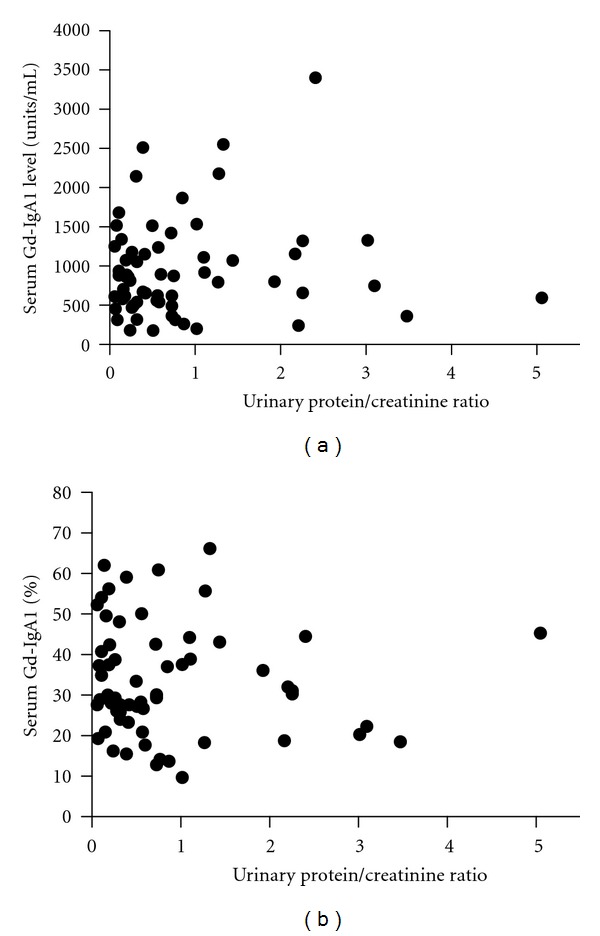
Random urinary protein/creatinine ratios. (a) Plotted against serum Gd-IgA1 levels. Spearman's rank correlation is *r* = 0.05, *P* = 0.72. (b) Plotted against percent Gd-IgA1 levels. Spearman's rank correlation is *r* = −0.11, *P* = 0.38.

**Figure 3:**
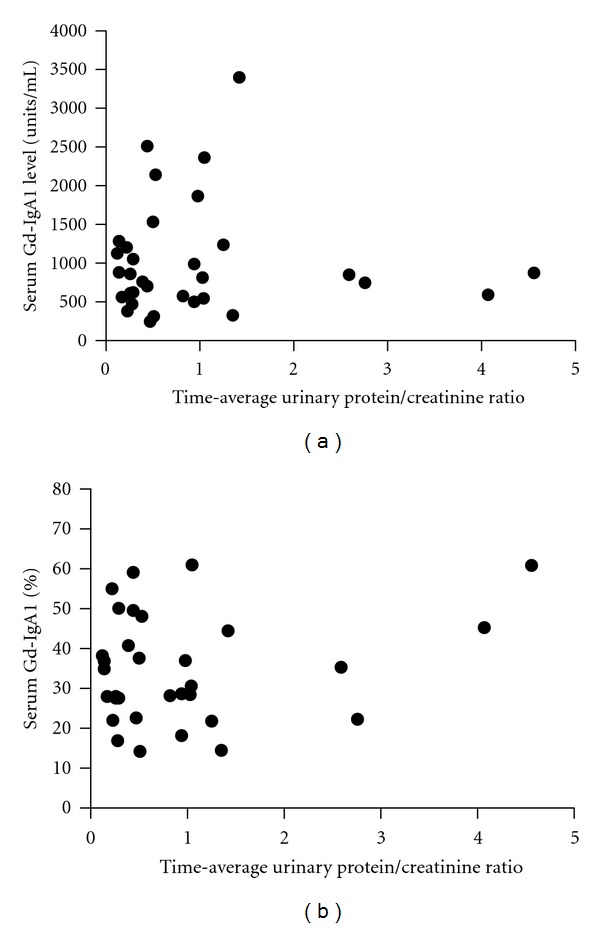
Time-average urinary protein/creatinine ratios. (a) Plotted against average serum Gd-IgA1 levels. Spearman's rank correlation is *r* = 0.06, *P* = 0.75. (b) Plotted against serum percent Gd-IgA1 levels. Spearman's rank correlation is *r* = 0.04, *P* = 0.87.

Spearman's correlation coefficients were also calculated for serum Gd-IgA1, percent Gd-IgA1, age at biopsy, and length of follow-up versus initial UPCR and TA-UPCR. For patients having two or more measurements for serum Gd-IgA1 and percent Gd-IgA1, the mean of these measurements was used for the calculation of correlation coefficients versus TA-UPCR. Again, all correlations were determined to be statistically insignificant and are as follows: serum Gd-IgA1 versus UPCR (*r* = 0.05, *P* = 0.72; Figure 2(a)), percent serum Gd-IgA1 versus UPCR (*r* = −0.11, *P* = 0.38; Figure 2(b)), serum Gd-IgA1 versus TA-UPCR (*r* = 0.06, *P* = 0.75; Figure 3(a)), percent serum Gd-IgA1 versus TA-UPCR (*r* = 0.04, *P* = 0.87; Figure 3(b)), age at time of biopsy versus TA-UPCR (*r* = 0.18, *P* = 0.32), and length of follow-up versus TA-UPCR (*r* = 0.10, *P* = 0.60). Of the 3 patients who progressed to ESRD, all had TA-UPCR above 1.0 and two had serum Gd-IgA1 levels above the 95th percentile for healthy children.

**Figure 4:**
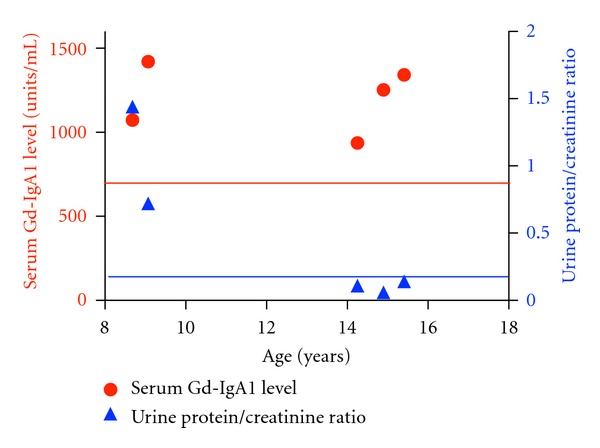
Serum Gd-IgA1 levels (red circles) and concomitant urinary protein/creatinine ratios are plotted for the patient having the most serum Gd-IgA1 determinations. The red line represents the 95th percentile for serum Gd-IgA level in healthy pediatric controls, and the blue line represents the upper limit of normal for urinary protein/creatinine ratio of 0.2. This time course is of interest in that the initial value was at presentation with gross hematuria and the last three values were obtained during clinical remission (normal urinalysis and estimated GFR).

UPCR and serum Gd-IgA1 levels were plotted against age for the subject with the longest period of serial serum Gd-IgA1 sampling (Figure 4). This clinical course is of interest in that the blood sample for measurement of the first level was obtained during an episode of gross hematuria at time of diagnosis and the last three blood samples were collected during clinical remission (urinalysis, UPCR and serum creatinine concentration all normal).

## 4. Discussion

Remission of proteinuria is an important predictor of renal survival. Analysis of 542 adult patients with IgAN in the Toronto Glomerulonephritis Registry showed that when treatment achieved a mean urinary protein excretion <1 g/day over the follow-up interval, the decline in glomerular filtration rate was markedly slower than that for the entire cohort [7]. Thus, magnitude of proteinuria is a useful surrogate marker of outcome in IgAN patients detected early in the course of disease when renal clearance function is normal.

Serum Gd-IgA1 level can be expressed as an absolute level or as a percentage of total serum IgA. In our earlier cohort of 153 adults with IgAN, the absolute serum level was a better diagnostic marker than percent Gd-IgA1, but neither Gd-IgA1 (*r* = −0.128, *P* = 0.211) [14] nor percent Gd-IgA1 (*r* = −0.022,  *P* = 0.788) (previously unpublished data) correlated significantly with UPCR. In that study, only 24 subjects were sampled within 8 weeks of biopsy for measurement of the serum Gd-IgA1 level [14]. However, the median serum Gd-IgA1 level for those 24 subjects was similar to the median for 26 subjects who had levels measured at the last follow-up when the urinalysis and UPCR were normal.

In a Japanese cohort, however, there was no clinically significant difference in magnitude of proteinuria when subjects over the age of 16 years were stratified by serum Gd-IgA1 level above versus below the 90th percentile for healthy controls [15]. The urine protein excretion for the 20 subjects with serum Gd-IgA1 levels above the 90th percentile was 1.0 g/day versus 1.1 g/day for the 21 subjects with lower serum Gd-IgA1 levels. About 95% of the serum samples used for that study were obtained at the time of biopsy.

In a study by Camilla et al. [23], their cohort included adults and children with IgAN and a correlation between percent Gd-IgA1/IgA and contemporaneous UPCR was found to be marginally significant (*r* = 0.25, *P* = 0.03). The correlation improved when TA-UPCR for each patient was correlated against a single serum Gd-IgA1 level (*r* = 0.29, *P* = 0.007). Our failure to confirm this finding in a pediatric cohort is not easily explained. A possible basis is a difference in severity of disease. Our pediatric cohort had a higher percentage of patients with low TA-proteinuria.

Serum Gd-IgA1 and percent Gd-IgA1 levels are often elevated in first-degree relatives of patients with IgAN [15, 27, 28]. However, virtually all of these affected relatives had no clinical evidence for renal disease prior to or at the time of blood sampling [15, 27, 28]. In addition, careful examination of the values for healthy controls indicates that serum Gd-IgA1 levels are distributed in a nonparametric manner, such that at least 5% of the subjects had a level higher than predicted by a normal distribution [14, 15, 23].

Thus, the presence of serum Gd-IgA1 is necessary but not sufficient to precipitate the renal injury in IgAN [12]. In the study of Suzuki et al. [19], levels of IgG specific for Gd-IgA1 correlated with UPCR (*P* < 0.0001) and with the levels of IgA-IgG immune complexes normalized to urinary creatinine (*P* = 0.0082) in contemporaneously collected urine samples. This prognostic significance may be due to a requirement for the formation of immune complexes to sustain renal injury in IgAN. The serum Gd-IgA1 level seems to be more valuable as a marker for risk of disease rather than for prognosis [14].

## 5. Conclusion

In a cohort of pediatric patients with IgAN, we failed to confirm a recently described association between the magnitude of proteinuria and percent serum Gd-IgA1/IgA [21]. This finding is similar to that in our initial North American cohort and a Japanese cohort of adults [14, 15]. Thus, the weight of the evidence to date fails to support a relationship between the serum Gd-IgA1 level and severity of proteinuria. This clinical expression of disease in IgAN is likely influenced by other factors or hits, such as levels of circulating antibodies specific for Gd-IgA1, level and/or composition of Gd-IgA1-containing immune complexes, or other factors that influence mesangial inflammation.

## Figures and Tables

**Table 1:** Clinical and demographic features of individual subjects (incident cases).

Subject	Race/gender	Presentation	Age at	estGFR	Urinary protein/	BMI	SBP/DBP	Serum Gd-IgA1
			biopsy	(mL/min/1.73 m^2^)	creatinine ratio	percentile	percentile	level (units/mL)
A1	C/M	Microhematuria,	17.6	59	1.17	98	95–99/<50	262
		proteinuria						
M2	C/M	Isolated proteinuria	13.8	78	1.24	26	95–99/50–90	889
M15	C/F	Gross hematuria	11.2	80	1.31	<1	<50/50–90	1076
M16	C/F	Gross hematuria	8.7	75	1.00	57	50–90/<50	1073
M17	C/M	Gross hematuria	13.4	108	3.10	11	50–90/50–90	1534
M18	C/F	Recurrent gross hematuria	12.5	107	2.96	17	>99/<50	1155
M19	AA/F	Gross hematuria,	15.9	49	2.97	52	<50, <50	3401
		rapidly progressive						
		glomerulonephritis						
M21	AA/M	Isolated proteinuria	5.1	94	3.58	96	<50, 50–90	747
M22	C/M	Gross hematuria,	8.6	75	1.33	49	95–99/50–90	803
		acute kidney injury						
M26	AA/M	Gross hematuria	8.8	120	3.70	30	90–95/50–90	470
M28	C/F	Gross hematuria,	16.7	63	1.36	40	90–95/50–90	1110
		proteinuria						
M29	C/M	Gross hematuria	12.8	96	0.95	41	>99/95–99	659
M30	C/M	Gross hematuria,	15.0	61	2.89	63	90–95/50–90	364
		acute kidney injury						
M31	C/M	Gross hematuria	16.2	84	0.25	89	50–90/50–90	2513
T2	C/M	Microhematuria, proteinuria,	17.8	40	1.27	44	95–99/50–90	796
		Chronic kidney						
		disease stage 3						

AA: African-American; BMI: body mass index; C: Caucasian; estGFR, estimated glomerular filtration rate; DBP: diastolic blood pressure; SBP: systolic blood pressure; microhematuria, >5 RBC/high powered field.

**Table 2:** Clinical and demographic features of individual subjects (prevalent cases).

Subject	Race/gender	Presentation	Age at biopsy	estGFR (mL/min/1.73 m^2^)	Urinary protein/ creatinine ratio	BMI percentile	SBP/DBP percentile	Serum Gd-IgA1 level (units/mL)
A2	AA/F	Gross hematuria,	15.9	98	1.13	99	95–99/95–99	1320
		proteinuria						
A3	C/F	Gross hematuria,	6.8	82	5.00	54		753
		nephrotic syndrome						
K1	C/M	Gross hematuria	11.2	87	1.40	99	50–90/50–90	704
K2	C/M	Microhematuria,	13.2	138	0.41	99	>99/50–90	919
		proteinuria						
M1	AA/M	Microhematuria,	9.3	108	300 mg/dL*	97	95–99/50–90	1328
		proteinuria						
M3	C/M	Isolated proteinuria,	13.0	50	4.12	95	95–99/50–90	594
		chronic kidney disease						
		stage 3						
M4	C/M	Recurrent gross hematuria,	14.5	79	0.72	93	90–95/50–90	1867
		acute kidney injury						
M5	C/F	Gross hematuria	11.6	79	3.61	61	95–99/50–90	363
M6	C/M	Gross hematuria	11.6	98	255 mg**	74	90–95/95–99	2177
M7	AA/F	Gross hematuria,	10.4	70	3.29	33	50–90/50–90	315
		nephrotic syndrome,						
		acute kidney injury						
M8	C/M	Gross hematuria	17.4	67	0.44	94	90–95/50–90	1054
M9	C/M	Recurrent gross hematuria	16.5	100	0.41	76	90–95/50–90	861
M10	AA/M	Recurrent gross hematuria	7.0	102	0.64	90	95–99/50–90	563
M11	AA/F	Gross hematuria	5.5	95	30 mg/dL*	93	50–90/<50	882
M12	C/M	Gross hematuria	8.5	87	0.04	15	50–90/50–90	612

AA: African-American; BMI: body mass index; C: Caucasian; estGFR: estimated glomerular filtration rate; DBP: diastolic blood pressure; SBP: systolic blood pressure.

*Amount of protein by urinary dipstick;

**Amount of protein in 24 hours.

**Table 3:** Clinical and demographic features of cohorts.

	LBCH cohort	Other Cases
	*n* = 31	*n* = 9
Incident cases	13	2
Prevalent cases	18	7
Male	21	5
Female	10	4
Caucasian	21	8
African-American	10	1
Age at biopsy, yrs	11.5 ± 4.7	12.4 ± 4.9
Follow-up after biopsy all	4.2 ± 2.4	1.9 ± 2.0
patients, yrs, mean ± SD		
CKD5 at last follow-up	3	0
TA-UPCR data, patients	29	3
Follow-up after biopsy for	4.2 ± 2.4	3.1 ± 2.1
TA-UPCR patients, yr, mean ± SD		
TA-UPCR ≥ 1.0	9	1
TA-UPCR ≥ 0.5, < 1.0	7	0
TA-UPCR < 0.5	13	2

CKD5: chronic kidney disease stage 5; LBCH: Le Bonheur Children's Hospital; SD: standard deviation; TA-UPCR: time-average urinary protein/creatinine ratio; yrs: years.
